# Conjugated phthalocyanine-based framework as artificial SEI for over 400 Wh kg^−1^ lithium-metal battery

**DOI:** 10.1093/nsr/nwae443

**Published:** 2024-12-09

**Authors:** Ying Zang, Peng Peng, Fei Pei, Run-Han Li, Lin Wu, Di-Qiu Lu, Yi Zhang, Kai Huang, Yue Shen, Yun-Hui Huang, Ya-Qian Lan

**Affiliations:** School of Chemistry, South China Normal University, Guangzhou 510006, China; Henan Key Laboratory of Crystalline Molecular Functional Materials, Henan International Joint Laboratory of Tumor Theranostical Cluster Materials, Green Catalysis Center, College of Chemistry, Zhengzhou University, Zhengzhou 450001, China; State Key Laboratory of Materials Processing and Die & Mould Technology, School of Materials Science and Engineering, Huazhong University of Science and Technology, Wuhan 430074, China; School of Chemistry, South China Normal University, Guangzhou 510006, China; State Key Laboratory of Materials Processing and Die & Mould Technology, School of Materials Science and Engineering, Huazhong University of Science and Technology, Wuhan 430074, China; School of Chemistry, South China Normal University, Guangzhou 510006, China; State Key Laboratory of Materials Processing and Die & Mould Technology, School of Materials Science and Engineering, Huazhong University of Science and Technology, Wuhan 430074, China; State Key Laboratory of Materials Processing and Die & Mould Technology, School of Materials Science and Engineering, Huazhong University of Science and Technology, Wuhan 430074, China; State Key Laboratory of Materials Processing and Die & Mould Technology, School of Materials Science and Engineering, Huazhong University of Science and Technology, Wuhan 430074, China; State Key Laboratory of Materials Processing and Die & Mould Technology, School of Materials Science and Engineering, Huazhong University of Science and Technology, Wuhan 430074, China; School of Chemistry, South China Normal University, Guangzhou 510006, China

**Keywords:** conjugated phthalocyanine, artificial SEI, high energy density, lithium-metal battery

## Abstract

High-voltage lithium-metal batteries (HVLMBs) are appealing candidates for next-generation high-energy rechargeable batteries, but their practical applications are still limited by the severe capacity degradation, attributed to the poor interfacial stability and compatibility between the electrode and the electrolyte. In this work, a 2D conjugated phthalocyanine framework (CPF) containing single atoms (SAs) of cobalt (CoSAs-CPF) is developed as a novel artificial solid–electrolyte interphase (SEI) in which a large amount of charge is transferred to the CPF skeleton due to the Lewis acid activity of the Co metal sites and the strong electron-absorbing property of the cyano group (−CN), greatly enhancing the adsorption of the Li^+^ and regulating the Li^+^ distribution toward dendrite-free LMBs, which are superior to most of the reported SEI membranes. As a result, the Li||Li symmetrical cell with CoSAs-CPF-modified Li anodes (CoSAs-CPF@Li) exhibits a low polarization with an area capacity of 1.0 mAh cm^−2^ over 3500 h. The LiFePO_4_ (LFP) ||CoSAs-CPF@Li (LFP: 20 mg cm^−2^) delivers an ultra-long cycling life of ≤1000 cycles with a high capacity retention of 98.6%. Remarkably, the high-voltage LiNi_0.8_Co_0.1_Mn_0.1_O_2_||Li@CoSAs-CPF (NCM811: 10 mg cm^−2^) demonstrates a long cycling life of >800 cycles with a high capacity retention of 80%. Meanwhile, *in situ* ultrasonic transmission technology confirms the admirable ability of artificial CoSAs-CPF SEI to stabilize the Li-anode interface in pouch cells during cycling. Remarkably, the NCM811||Li@CoSAs-CPF pouch cell exhibits an energy density of 421 Wh kg^−1^ and keeps 130 cycles with a low electrolyte/capacity ratio of 2.5 g Ah^−1^. The strategy of constructing the CoSAs-CPF-reinforced Li anode provides a promising direction for high-energy-density HVLMBs with long cycling stability.

## Teaser

A two-dimensional conjugated phthalocyanine framework containing single cobalt atoms (CoSAs-CPF) is developed as a promising artificial solid electrolyte interphase (SEI) membrane. It delivers an outstanding performance on stabilizing the traditional anode-electrolyte interface, improving the capacities and rate capabilities of lithium metal batteries.

## INTRODUCTION

The development of portable electronic devices and electric vehicle technology has created a huge demand for high-energy-density energy-storage systems. Lithium-metal batteries (LMBs) have been accepted as one of the most appealing candidates to meet the increasing demand for high-energy-density energy-storage devices, due to the high theoretical capacity (3860 mAh g^−1^) and low redox potential (3.04 V vs standard hydrogen electrode) of the Li-metal anode [1–8], especially when coupled with high-voltage (LiNi_0.8_Co_0.1_Mn_0.1_O_2_ (NCM811)) and/or high-capacity (S and O_2_) cathodes [5,9–11]. Unfortunately, the fast capacity degradation of LMBs and potential safety hazards, which are caused by the poor stability of the solid–electrolyte interphase (SEI) and uncontrolled Li-dendrite growth/pulverization, have limited further commercialization of LMBs [2,4,12–15].

To solve these issues, multitudinous tactics have been exploited to reinforce the SEI layer by using various electrolyte additives or novel lithophilic current collectors [2,4,12,16,17]. Although these strategies have improved the uniform deposition of Li and the cyclability of LMBs to some extent, *in situ*-generated non-uniform organic–inorganic hybrid SEIs are still insufficient to inhibit the growth of needlelike dendrites. In this case, an artificial SEI film is considered as a highly effective way to solve interface problems of the Li anode due to (i) the chemical components of the artificial SEI film are uniform, which is conducive to the uniform diffusion of lithium metals; (ii) it can be rationally designed to obtain a better morphological structure and improve the electrochemical performance of the electrode interface and (iii) it can effectively isolate the contact between the electrolyte and the electrode material, which reduces the electrolyte reaction, thus improving the coulomb efficiency [11,18–23]. It is worth noting that the simultaneous fulfillment of all the above requirements is challenging. In recent years, graphene films, Al_2_O_3_ and lithium alloys have been widely used as a stable SEI film to isolate the unanticipated side reactions and protect the Li anode, and have also witnessed significant development for improving the interfacial stability of LMBs [24–29]. Although some progress has been made, practical application of LMBs are still limited by how to achieve better chemical compatibility, more substantial Li-ion transport efficiency and more uniform lithium-ion deposition.

Conjugated phthalocyanine frameworks (CPFs) with unique structural flexibility [30–33], excellent flexibility/elasticity [34,35] and good physicochemical stability [35,36] show enormous potential for constructing artificial SEIs: (i) the highly organic component composition exhibits excellent chemical compatibility/wettability with the electrolyte; (ii) the excellent flexibility and high elasticity guarantee the mechanical robustness of the SEI; (iii) CPFs are functionally tailorable, which is conducive to the realization of a faster ionic conduction [20,37,38]; (iv) the abundant functional coordination centers can uniformly provide metal sites with catalytic activity so, in the conjugated skeletons with a high degree of uniform dispersion of single metal sites, the electron-rich state around the metal atoms can optimize the Li^+^ local coordination environment and promote Li^+^ rapid migration, which is thought to potentially have a good inducing effect on the homogeneous deposition of lithium metals [38–41]. Notably, there are almost no 2D fully π-conjugated CPFs that have been reported as artificial SEI layers to date.

Herein, we developed a pyrolysis-free route to synthesize a series of 2D conjugated phthalocyanine frameworks that contained different metal single atoms (MSAs, including FeSAs, CuSAs, NiSAs, CoSAs) that were stabilized effectively in the ultra-thin 2D structure, which can guarantee higher atomic utilization and provide a more efficient charge-transfer pathway. The mild synthetic route effectively reduces the agglomeration of MSAs-CPF and guarantees the utilization of the metal sites. More importantly, the Lewis acid activity of the MSA sites and the strong electron-withdrawing property of the cyano groups promoted a large amount of charge to be transferred to the CPFs skeleton, which effectively enhanced the adsorption of the Li^+^ and suppressed Li dendrite. Meanwhile, MSAs-CPF-based flexible pack batteries also show a longer operating life and higher capacity retention. By calculation, it is found that a phthalocyanine framework containing single cobalt atoms (CoSAs-CPF) shows a significant advantage in the adsorption and migration process of lithium ions, which keeps a high consistency with the subsequent experiments. A Li||Li symmetrical cell with CoSAs-CPF-modified Li anodes exhibited a low polarization with an area capacity of 1.0 mAh cm^−2^ for 3500 h. The CoSAs-CPF anode endows the high-loading LFP cathode (20 mg cm^−2^) with a long cycling life of ≤1000 cycles. Notably, the NCM811|CoSAs-CPF@Li cells can deliver a stable cycling life of 800 and 450 cycles with a loading of 10 and 20 mg cm^−2^, respectively. By using *in situ* ultrasonic imaging technology, the deterioration of the Li-anode interface in the pouch cells during cycling can also be nondestructively shown. This is one of the pioneering systems to achieve the remarkable performance of high-voltage LMBs (HVLMBs). The strategy of constructing the CoSAs-CPF-reinforced Li anode provides a promising direction for long-cycling-life HVLMBs.

## RESULTS AND DISCUSSION

**Figure 1.**
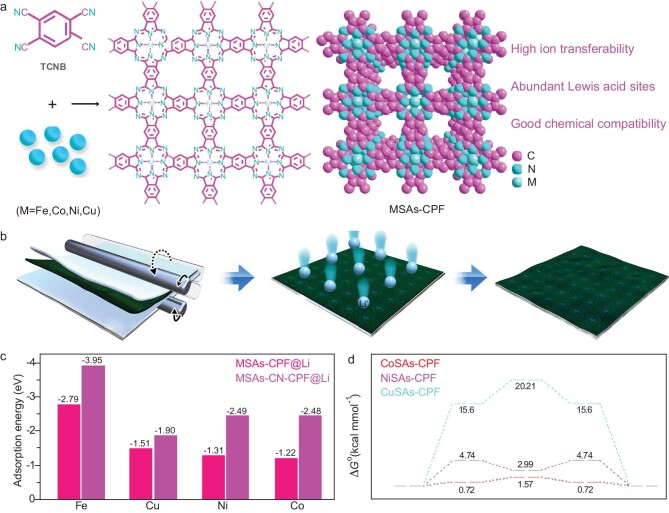
Structural design and comparison of lithiophilic properties of MSAs-CPF materials. (a) Schematic illustration of MSAs-CPF (M = Fe, Co, Ni, Cu). TCNB stands for 1,2,4,5-tetracyanobenzene. (b) Schematic illustration of the fabrication processes for MSAs-CPF@Li and Li deposition processes for MSAs-CPF@Li in lithium-metal batteries. (c) Binding energy of Li atoms on MSAs-CPF and MSAs-CN-CPF (CN stands for cyanide). (d) The migration-energy barriers of Li on CoSAs-CPF, NiSAs-CPF and CuSAs-CPF.

The conjugated quasi-phthalocyanine framework MSAs-CPF with single metal sites was synthesized by using a solvothermal method according to our previous reports [30,31] (Fig. 1a). The MSAs-CPF was then dispersed in anhydrous *N,N*-dimethylformamide to form a uniform mixed solution of MSAs-CPF nanosheets (MSAs-NSs) after intense ultrasonic treatment. As shown in Fig. 1b, a facile roll-pressing process was carried out to transfer the MSAs-NSs onto the Li surface after the MSAs-NSs were filtrated on the polypropylene (PP) membrane and the MSAs-NSs@Li was finally fabricated. The homogeneous nanosheet structure, good ionic conductivity and uniformly distributed monatomic M active sites can regulate the uniform disposition of Li and generate a stable LiF-rich interface to effectively inhibit the growth of lithium dendrites and protect the Li anode.

To investigate the adsorption effect of Li on the different metal centers-based conjugated quasi-phthalocyanine framework (Fig. 1a), the evaluation index of the adsorption-energy and migration-energy barriers were obtained by using charge decomposition analysis and density functional theory (DFT) calculations. Differential charge density maps showed the electron density distribution between the Li atoms and the MSAs-CPF, and the charge limitedly accumulated in the skeleton, clearly indicating that the adsorption process was accompanied by Li–M–N charge transfer (Fig. S1). Subsequently, the adsorption energy of a Li atom adsorbing on the surfaces of the MSAs-CPF was determined by using DFT. As shown in Fig. 1c, all of the metal sites show a relatively strong adsorption-energy effect toward Li, indicating that all these metals are lithophilic. Furthermore, the adsorption energies of Li^+^ at the peripheral cyanide sites were also investigated. As shown in Fig. 1c, the peripheral cyanide sites showed much higher adsorption energy, definitely indicating that the Li^+^ transfer at the peripheral cyanide sites was more difficult than that at the metal sites. Thus, the Lewis acid metal sites in the polyphthalocyanine framework are ideal adsorption sites for lithium ions.

A further analysis revealed that the CoSAs-CPF-based system presents a relatively low adsorption energy of −1.22 eV compared with −2.79 eV of FeSAs-CPF, −1.51 eV of CuSAs-CPF and −1.31 eV of NiSAs-CPF. This suggests that Li^+^ has a strong binding ability on the surface of the FeSAs-CPF and the effective migration of Li^+^ cannot be completed; however, CuSAs-CPF, NiSAs-CPF and CoSAs-CPF adsorb Li^+^ efficiently and guarantee the migration process. In order to be more explicit, a calculation model based on monocyanophthalocyanine instead of CPF was taken to illustrate the migration-energy barriers of Li^+^ on the CuSAs-CPF, NiSAs-CPF and CoSAs-CPF surfaces through a series of structural Gibbs energy calculations (Fig. 1d and Figs S2–S4). By analysing the migration potential energy of Li^+^, we could conclude that its migration is more likely to occur in the direction of M–N ring→pyrrole ring→benzene ring. In addition, when the potential energies of the Li^+^ migration on the surface of the Co (−1.22 eV), Ni (−1.31 eV) and Cu (−1.51 eV) centers were compared, the energy to be overcome for Li^+^ migration on the surface of the Co centers was found to be relatively low (1.57 kcal/mol), which suggests that the surface migration of Li^+^ is most thermodynamically favorable when the center metal is Co (Figs S5–S7). It is noteworthy that the polymerization of bis-phthalocyanine shows similar trends to those of monocyanophthalocyanine, further proving that the outstanding Li^+^ transport and performance in the CoSAs-CPF system could be ascribed to the Co sites. The above results indicate that MSAs-CPFs are ideal substrates for the adsorption of lithium. Benefitting from the effect of different Lewis acid metal sites, CoSAs-CPF is more favorable for the migration of lithium, so we synthesized CoSAs-CPF (NiSAs with a very similar results as a comparison), conducted the detailed structural characterization and employed it as the artificial SEI film of LMBs.

**Figure 2.**
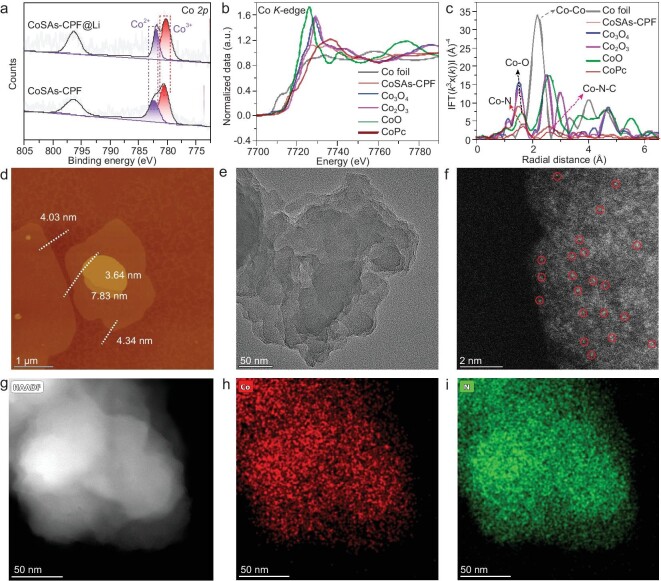
Structural characterization of CoSAs-CPF. (a) High-resolution Co *2p* spectra of CoSAs-CPF; the black curves are the fitted data while the gray lines are the experimental results. (b) Normalized X-ray absorption near edge structure (XANES) and (c) radial structure functions (RSFs) of the Co *K*-edge obtained by Fourier transformation k3-weighted extended x-ray absorption fine structure (EXAFS) results, with the Co foil, Co_2_O_3_, Co_3_O_4_ and CoPc serving as references. (d) AFM image of CoSAs-CPF. (e) TEM image of CoSAs-CPF. (f and g) HAADF-STEM image and (h and i) EDS-mapping results of CoSAs-CPF.

The characteristic peaks located at approximately 1547, 1697, 1711 and 1757 cm^−1^ in the Fourier transform infrared spectra (Fig. S8) represent that the specific macrocyclic structure of phthalocyanine was successfully constructed. In addition, the carbon structure of CoSAs-CPF was further confirmed by using ^13^C solid-state nuclear magnetic resonance (Fig. S9). The coordination environment of Co such as the valence state, coordination status and bonding configurations were identified in depth by using the X-ray photoelectron spectra (XPS) and the synchrotron-based X-ray absorption near edge structure (XANES) spectra (Fig. 2a–c). By comparison with those of the corresponding reference samples (Co foil, Co_2_O_3_, Co_3_O_4_ and cobalt phthalocyanine (CoPc)), the Co K-edge XANES curves (Fig. 2b) showed that the pre-edge profiles of CoSAs-CPF are similar to that of CoPc and they only have a slight difference in intensities, indicating the existence of the homogenous state of Co and the formation of Co–Nx coordination in CoSAs-CPF. As revealed by the extended X-ray absorption fine structure (EXAFS) spectra, the dominant peak that was generated by the Co–O (CoO as the reference) and Co–N species (CoPc as the reference) at ∼1.62 Å demonstrated a similar absorption position to CoPc (Fig. 2c), implying the presence of N-coordinated single Co atom sites.

Moreover, the absence of a peak at 2.17 Å belonging to the Co–Co metallic bond in CoSAs-CPF further validates the atomic dispersion of the Co atoms (Fig. 2c). Meanwhile, the prominent scattering path signal [χ(k), χ(R)] of the Co–N bonding of CoSAs-CPF in the first coordination shell is located at [3.79, 1.25], while a subtle scattering path signal of Co–C in the second coordination shell is located at [4.41, 2.23], which can be found in the wavelet transform of the χ(k) spectra (Fig. S10). Furthermore, the characteristic scattering path signal of the Co–Co bonding in the Cu foil located at [7.12, 2.14] was not observed in the CoSAs-CPF, indicating again the atomic dispersion of the Co atoms in CoSAs-CPF. Collectively, the coordinated environment of Co sites in CoSAs-CPF was well elucidated by the Co K-edge XANES curves, the related analysis results in R spaces and the wavelet transform of the χ(k) spectra, and the single-atomic states of Co atoms were also clearly proved.

Meanwhile, CoSAs-CPF showed a 2D slice morphology with a homogeneous distribution of different elementals from the atomic force microscope (AFM) (Fig. 2d), scanning electron microscope (SEM) (Fig. S11) and transmission electron microscope (TEM) images (Fig. 2e and Fig. S12). The thickness of the CoSAs-CPF was collected by using AFM, which was confirmed as being ∼3.5–8 nm (Fig. S13), corresponding to 10–18 atomic layers. Furthermore, as revealed by SEM and TEM images, it can be observed that there is no aggregation of metal particles on the surface of CoSAs-CPF, indicating that the Co species were uniformly distributed on CoSAs-CPF at the size of an atomic scale. Subsequently, the atomic structure of CoSAs-CPF was further explored by using a high-angle annular dark-field scanning transmission electron microscope (HAADF-STEM) (Fig. 2f–i). From the aberration-corrected HAADF-STEM image of CoSAs-CPF, it could be observed that there is also no trace of the presence of aggregated states (Fig. 2f). The elemental mapping also suggests that the coordinated Co atoms were completely atomically and highly uniformly distributed on the surface (Fig. 2g–i).

CoSAs-CPF@Li was fabricated by using a facile roll-pressing process (Fig. 1b). The optical photographs of CoSAs-CPF@PP (inset of Fig. S14) show that CoSAs-CPF was uniformly pumped onto the PP membrane and formed a dense film that was dark green (nearly black). Subsequently, the CoSAs-CPF@PP surface containing CoSAs-CPF was rolled onto 100 µm of Li metal to form CoSAs-CPF@Li, resulting in an artificial CoSAs-CPF SEI. Afterward, the CoSAs-CPF@Li was cut into small discs with a diameter of 16 mm for button-cell assembly and testing (Figs S15 and S16). It is worth noting that the contact angle of CoSAs-CPF@Li is significantly smaller than that of Li foil, which indicates that CoSAs-CPF has a better wettability to the electrolyte, and further indicates that the artificial CoSAs-CPF SEI membrane has good chemical compatibility (Fig. S17). In addition, the XPS of CoSAs-CPF@Li was measured and suggested good stability of Li (Fig. 2a, Figs S18 and S19). To further explore the induced behavior of different Lewis’ acid sites for Li deposition, isostructural NiSAs were synthesized and NiSAs-NSs@Li was prepared by using the same method. Afterward, detailed electrochemical tests were executed to evaluate its potential for practical application in LMBs.

**Figure 3.**
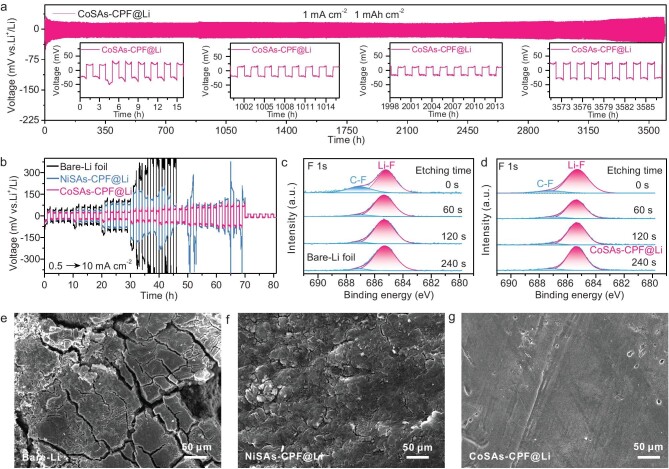
Electrochemical stability of the Li||Li symmetric cells. (a) Galvanostatic cycling profiles of CoSAs-CPF-modified Li||Li symmetric batteries with a current density of 1.0 mA cm^−2^ and areal capacity of 1.0 mAh cm^−2^. (b) Rate capabilities from 0.5 to 10 mA cm^−2^ of Li|Li symmetric batteries with CoSAs-CPF@Li, NiSAs-CPF@Li and bare-Li. Depth-profiled XPS data for (c) the cycled bare-Li anodes and (d) CoSAs-CPF@Li. Top-view SEM images of (e) the cycled bare-Li, (f) NiSAs-CPF@Li and (g) CoSAs-CPF@Li after rate cycles.

The constant current charging/discharging of Li||Li symmetric batteries were carried out to evaluate the effect of artificial CoSAs-CPF SEI on the Li deposition behavior and the cycling stability. As expected, under a constant current density of 1.0 mA cm^−2^ and areal capacity of 1.0 mAh cm^−2^ (Fig. 3a), the galvanostatic cycling profiles of symmetric batteries with artificial CoSAs-CPF SEI exhibited a prominent cycling stability for 3500 h and a low voltage fluctuation of ∼40 mV. Meanwhile, the rate capabilities from 0.5 to 10 mA cm^−2^ of CoSAs-CPF@Li, NiSAs-CPF@Li and bare-Li symmetric batteries were further tested with an area capacity from 0.5 to 10 mAh cm^−2^ (Fig. 3b). It can be clearly seen that the voltage polarization increases as the current density increases, and CoSAs-CPF@Li showed the slightest voltage polarization at different current densities, further highlighting its superior rate performance and promoting the homogeneous deposition of Li. The XPS of the cycled Li anodes with different times of etching (0, 60, 120 and 240 s) was performed to further analyse the chemical composition of the newly formed SEI. As is well known, the inorganic LiF improves the uniformity of Li deposition and facilitates the stability of the SEI, while the opposite is true for the inorganic phase C–F. As shown in the fitting consequences of Fig. 3c and d, the cycled CoSAs-CPF@Li showed significantly higher Li–F content and lower C–F content than that of the cycled bare-Li anode; the inorganic Li–F is uniformly distributed at each etching depth, suggesting that the stable artificial CoSAs-CPF SEI causes the TFSI^−^ anion to decompose preferentially into LiF, whereas the lower Li–F peak for the cycled bare-Li anode means that only a small amount of TFSI^−^ was reduced. In other words, CoSAs-CPF@Li delivered the ability to inhibit the further decomposition of electrolytes and keep the interfacial stability. After the rate cycles, the surface of cycled NiSAs-CPF@Li and bare-Li showed a severe crack and was uneven, which was caused by the uneven uniformity deposition of the Li^+^ and, on the contrary, the surface of the cycled CoSAs-CPF@Li remained relatively smooth, which was consistent with the rate performance (Fig. 3e–g).

**Figure 4.**
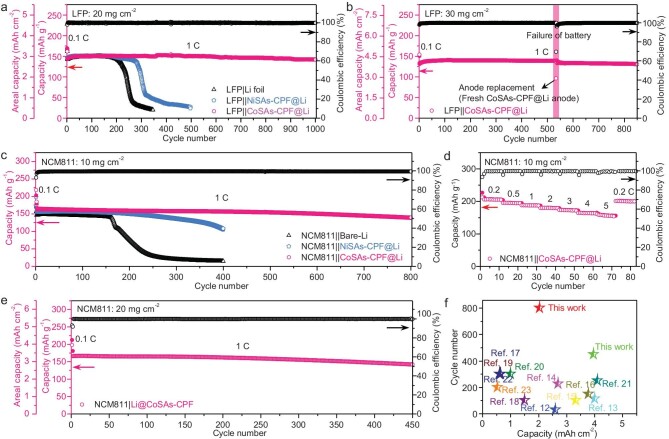
Electrochemical performance of the LFP||Li and NCM811||Li batteries. (a) Cycling performance of LFP||CoSAs-CPF@Li, LFP||NiSAs-CPF@Li and LFP||bare-Li (20 mg cm^−2^) at 1 C. (b) Cycling performance of LFP||CoSAs-CPF@Li (30 mg cm^−2^) at 1 C. (c) Cycling performance of NCM811||CoSAs-CPF@Li, NCM811||NiSAs-CPF@Li and NCM811||bare-Li (10 mg cm^−2^) at 1 C. (d) Rate performance of NCM811||CoSAs-CPF@Li (10 mg cm^−2^). (e) Cycling performance of NCM811||CoSAs-CPF@Li (20 mg cm^−2^) at 1 C. (f) Performance comparison of the coin-type batteries with the NCM811 cathodes.

LFP||CoSAs-CPF@Li batteries with high LFP loading (20 and 30 mg cm^−2^) were employed to explore the potential application and practical feasibility of the artificial SEI. As a result, the specific capacity of the CoSAs-modified batteries with an LFP loading of 20 mg cm^−2^ was as high as 144 mAh g^−1^ at 1 C with a coulombic efficiency of 99% and remained with almost no capacity degradation (98.6% capacity retention) after an ultra-long life of 1000 cycles (Fig. 4a). As a comparison, the bare-Li and NiSAs-modified Li anodes showed a similar initial capacity, but the capacity sharply dropped after 200 and 280 cycles, respectively. This is undoubtedly due to the uncontrollable growth of the Li dendrite of LFP||NiSAs-CPF@Li and LFP||bare-Li that was breaking out to different extents. It is also exciting to note that, when an ultra-high LFP loading of 30 mg cm^−2^ was executed, the LFP||CoSAs-CPF@Li battery system contributed a capacity of 133 mAh g^−1^ and only showed a slight capacity-decay tendency after continuing for 533 cycles (Fig. 4b). These batteries were then disassembled and replaced with fresh CoSAs-CPF@Li to assemble fresh batteries and the restarted battery could still maintain a stable life for 850 cycles under the same conditions. Such excellent results under ultra-high LFP loadings are uncommon and have been superior to those of most reports (Tables S1 and S2); they further reflect that the designed artificial SEI (CoSAs-CPF) can effectively induce the uniform deposition of Li and inhibit the decomposition of the electrolyte and the growth of Li dendrites even under an ultra-high LFP loading.

We further investigated the practical potential of CoSAs-CPF@Li for high-energy-density cells that are made of NCM811. The long-term cycling performance of NCM811||CoSAs-CPF@Li, NCM811||NiSAs-CPF@Li and NCM811||bare-Li (NCM811 loading: 10 mg cm^−2^) was evaluated. As shown in Fig. 4c, after activation at 0.1 C for two cycles, NCM811||CoSAs-CPF@Li shows an initial capacity of 166 mAh g^−1^ at 1 C, which is superior to those of NCM811||NiSAs-CPF@Li and NCM811||bare-Li (159 and 146 mAh g^−1^, respectively). It is worth noting that NCM811||CoSAs-CPF@Li also shows a prominent retention of ≤80% of the discharge capacity after 800 cycles and near-perfect coulombic efficiency (∼99.4%), signifying that the electrode materials were highly efficiently utilized and the efficiency of the charging/discharging was guaranteed. Conversely, NCM811||NiSAs-CPF@Li and NCM811||bare-Li possessed <80% capacity retention after 300 and 160 cycles, respectively. The discharge capacities of the NCM811||CoSAs-CPF@Li were 206, 196, 188, 180, 173, 164 and 157 mAh g^−1^ at current densities of 0.2, 0.5, 1, 2, 3, 4 and 5 C, respectively (Fig. 4d). Meanwhile, the capacity returned to the initial level when the rate was switched back to 0.2 C. More importantly, by increasing the loading of NCM811 to 20 mg cm^−2^ to meet the higher practical challenges (Fig. 4e), after activation at 0.1 C for one cycle, NCM811||CoSAs-CPF@Li produced an initial capacity of 166 mAh g^−1^ at 1 C, achieved outstanding stable cycling over 450 cycles at 1 C with 85.6% capacity retention and delivered high Li^+^ utilization with 99% coulombic efficiency. The performance was very competitive compared with other NCM811-based cathodes with modified Li anodes, as shown in Fig. 4f and Tables S1 and S2 [42–53].

**Figure 5.**
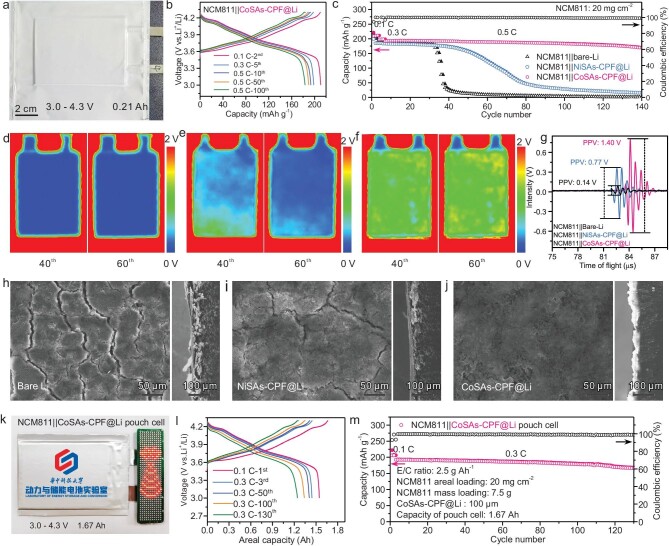
Electrochemical properties of NCM811||Li pouch cells. (a) Optical image, (b) charge/discharge curves and (c) cycling performance of the NCM811||CoSAs-CPF@Li pouch cell. *In situ* ultrasonic transmission images of (d) NCM811||bare-Li, (e) NCM811||NiSAs-CPF@Li and (f) NCM811||CoSAs-CPF@Li during the first 60 cycles. (g) Comparison of corresponding representative ultrasonic voltage signals. Top-view and cross-sectional SEM images of (h) bare-Li, (i) NiSAs-CPF@Li and (j) CoSAs-CPF@Li after 60 cycles. (k) Optical image of NCM811||CoSAs-CPF@Li pouch cell lighting an LED lamp. (l) Charge/discharge curves and (m) cycling performance of 1.67-Ah NCM811||CoSAs-CPF@Li pouch cell.

The digital photograph shows that the electrode size of the pouch cell is 6 cm × 8 cm (Fig. 5a). The cycling performance of the NCM811||CoSAs-CPF@Li pouch cell was investigated (Fig. 5b and c). The NCM811||CoSAs-CPF@Li cell with a high-loading cathode (NCM811: 20 mg cm^−2^) delivered a high discharge capacity of 210 mAh g^−1^ (4.2 mAh cm^−2^) at 0.1 C. The discharge capacity in the first cycle at 0.5 C was as high as 190 mAh (198 mAh g^−1^) and the pouch cell exhibited a discharge capacity of 169 mAh g^−1^ after 140 cycles with 85.4% capacity retention. This provides a promising strategy for the design of high-energy-density and high-voltage LMBs. The ultrasonic imaging technique is an interface-sensitive method to test the wetting state and gas generation in pouch cells. The ultrasonic signal will be attenuated significantly more in a vacuum or gas at the electrode interface than in solids. The ultrasonic signal changes from red to blue, indicating that the signal strength changes from high to low.

The NCM811||bare-Li and NCM811||NiSAs-CPF@Li pouch cells showed inhomogeneity after cycling and the image gradually turned blue with the increased Li-dendrite cycling number, explaining the growth and severe pulverization of Li dendrites (Fig. 5d and e). However, the interface in the NCM811||CoSAs-CPF@Li pouch remained in the initial state with the cycle number increasing, indicating the existence of an artificial SEI film that stabilizes the lithium-metal interface and prolongs the cycle life of the high-voltage battery (Fig. 5f). The corresponding representative ultrasonic signals are shown in Fig. 5g and the average peak–peak amplitude values between the recorded maximum and minimum signals of NCM811||bare-Li, NCM811||NiSAs-CPF@Li and NCM811||CoSAs-CPF@Li after 60 cycles were 0.14, 0.77 and 1.40 V, respectively. The unstable interface of the bare-Li metal caused the uncontrollable growth of lithium dendrites. The top-view SEM images of the cycled Li anodes were collected by disassembling the NCM811||Li pouch cells after 60 cycles. Li dendrites on the cycled bare-Li foil and NiSAs-CPF@Li had apparently grown. The surface of the Li metal with NiSAs-CPF@Li appeared to have many cracks and powder (Fig. 5h and i). The cycled CoSAs-CPF@Li appears to be relatively flat (Fig. 5j), so the high mechanical strength of the SEI and the uniform Li^+^ flux effectively suppressed the growth of lithium dendrites, creating a stable contact interface. More importantly, a high-capacity (1.67 Ah) multi-layer pouch cell was assembled (Fig. 5k), further verifying the key role of protection of the Li-metal anode (CoSAs-CPF@Li) to extend the cycle life. Under more demanding conditions, the electrolyte/capacity ratio (E/C) and the thickness of the Li anode were set to 2.5 g Ah^−1^ and a total NCM811 loading of 7.5 g; the pouch cell exhibited a high charge capacity of 1.67 Ah at 0.1 C in the first cycle with a high initial coulombic efficiency of 92.9% and, after cycling steadily at 0.3 C for 130 cycles, a discharge capacity of 166 mAh g^−1^ was maintained, with a high capacity retention of 86.5% (Fig. 5l and m).

## CONCLUSION

In summary, a promising artificial SEI membrane was successfully explored and prepared by using an atomically dispersed CoSAs-CPF to truly demonstrate the concept of the adsorption of Li^+^ and to effectively suppress Li dendrites. The mild route of the pyrolysis-free synthetics for CoSAs-CPF not only effectively reduced the formation of agglomeration, but also maintained the integrity of the structure, providing a new insight for the preparation of an artificial SEI with outstanding performance by stabilizing the traditional anode–electrolyte interface. Benefitting from highly atomically uniformly dispersed cobalt Lewis’ acid sites, excellent electron-withdrawal properties, good charge-transferring ability and stretchable flexibility, the CoSAs-CPF-modified SEI has become an ideal platform for the construction of high-performance HVLMBs. As expected, the capacities, rate capabilities and especially the cyclic stability of the batteries were significantly enhanced and superior to most of the reported SEI membranes. Given the diversity of CPF structures, chemically modifiable functional groups on organic linkers and the functional modulation of CPF properties, it is believed that the concept of a functionalized CPFs-based artificial SEI will push forward the future development of high-energy-density HVLMBs.

## Supplementary Material

nwae443_Supplemental_File
