# Supplementary material for: Conjugated phthalocyanine-based framework as artificial SEI for over 400 Wh kg−1 lithium-metal battery
Source: Natl Sci Rev. 2024 Dec 9;12(2):nwae443. doi: 10.1093/nsr/nwae443 (PMC11740503; doi:10.1093/nsr/nwae443)
Supplement: nwae443_Supplemental_File [file nwae443_supplemental_file.pdf]

Supporting Information

Conjugated Phthalocyanine Based Framework as Artificial SEI for over 400 Wh kg^{-1} Lithium Metal Battery

Ying Zang[1], Peng Peng[3], Fei Pei[2*], Run-Han Li[1], Lin Wu[2], Di-Qiu Lu[1], Yi Zhang[2], Kai Huang[2], Yue Shen[2], Yun-Hui Huang[2*], Ya-Qian Lan[1*]

1 School of Chemistry, South China Normal University, Guangzhou 510006, P. R. China.

2 State Key Laboratory of Materials Processing and Die & Mould Technology, School of Materials Science and Engineering, Huazhong University of Science and Technology, Wuhan, 430074, P. R. China

3 Henan Key Laboratory of Crystalline Molecular Functional Materials, Henan International Joint Laboratory of Tumor Theranostical Cluster Materials, Green Catalysis Center, and College of Chemistry, Zhengzhou University, Zhengzhou, 450001, P. R. China

Materials and reagents

All analytical grade chemicals are analytical grade were purchased through commercial suppliers (Aladdin and Sigma-Aldrich), and used without further purification. Water used in this work was purified using the Milli-Q purification system.

Experimental Section

Synthesis of CoSAs-CPF and NiSAs-CPF: Benzene-1,2,4,5-tetracarbonitrile (TCNB) was used as the monomers via a solvothermal process (180 ^{o}C for 4 days) with 1,8-Diazabicyclo(5,4,0)undec-7-ene (DBU) as the catalysts. Typically, TCNB (0.10 g, 0.560 mmol), cobalt or nickle chloride (0.280 mmol), and DBU (0.10 mL) were dissolved in 9 mL ethylene glycol and DMF (v:v = 9:1), the reaction was going on 24 h. After cooling to room temperature, the precipitate was collected by filtration, and washed with water. The solid was dried to afford CoSAs-CPF and NiSAs-CPF as a dark green powder (Yield: 74%).

Synthesis of NiSAs-CPF@Li and CoSAs-CPF@Li: The NiSAs-CPF or CoSAs-CPF nanosheets were first dispersed in *N*,*N*-Dimethylacetamide (DMAc) to form a homogeneous solution (The concentration is 0.5 mg mL^{-1}). Then, the dispersion was filtered through PP separator (The diameter is 5 cm) filtered through a piece of commercial PP to form NiSAs-CPF@PP or CoSAs-CPF@PP, The mass loading of NiSAs-CPF and CoSAs-CPF are 0.5 mg cm^{-2}. The NiSAs-CPF@Li and CoSAs-CPF@Li were obtained through roll-to roll fabrication system using a piece of $EMoS_2$@PP and Li foil. By roll-to-roll calendering, pre-manufactured NiSAs-CPF and CoSAs-CPF could be fully transfer-printed onto Li foil, and then cut into a disc diameter of 16 mm.

Characterization

Electrochemical measurement: NCM811 and LFP cathodes were prepared by following steps. The mixture of 0.9 g NCM811 (or LFP) powder, 50 mg (5wt%) PVDF (dispersed in N -methyl-2-pyrrolidone) and 50 mg Super P was stirred for 6 h to form a uniform slurry. Then the

slurry was casted on an Al foil and dried at 80 ℃ for 24 h under vacuum. The average mass loading of active material loading (NCM811 and LFP) were controlled at 10 mg cm^{-2} and 20 mg cm^{-2}. The CR2032 coin cells were assembled in an Ar-filled glove box (O_2 and H_2O < 0.1 ppm) using 1 M $LiPF_6$ in EC/EMC/DMC (1:1:1 in volume) with 5 wt% FEC (Fluoroethylene carbonate) as the electrolyte. NCM811||Li pouch cells (6 × 8 cm^2) were assembled in a dry room. The mass loading of NCM811 cathode is 20 mg cm^{-2}, Al-plastic film, nickel and Al cell tabs were and the were purchased from Canrd Technology Co. Ltd. Galvanostatic measurements for coin cells and pouch cells were conducted with a NEWARE battery test instrument at 26 ℃.

Computational details

All density functional theory (DFT) calculations are performed with Gaussian 16[1] in this work. All structures are fully optimized using def2-SVP[2] basis set and B3LYP functional[3] with Grimme's DFT-D3(BJ) empirical dispersion correction[4]. Vibrational frequencies are calculated using the same theoretical level to verify whether the optimized structures are in equilibrium states. To obtain more precise adsorption energies, the def2-TZVP basis set[2] is used to calculate the Gibbs free energy(ΔG°) based on the optimized structures. Charge decomposition analysis[5] (CDA) and electron density differences are evaluated by Multiwfn[6].

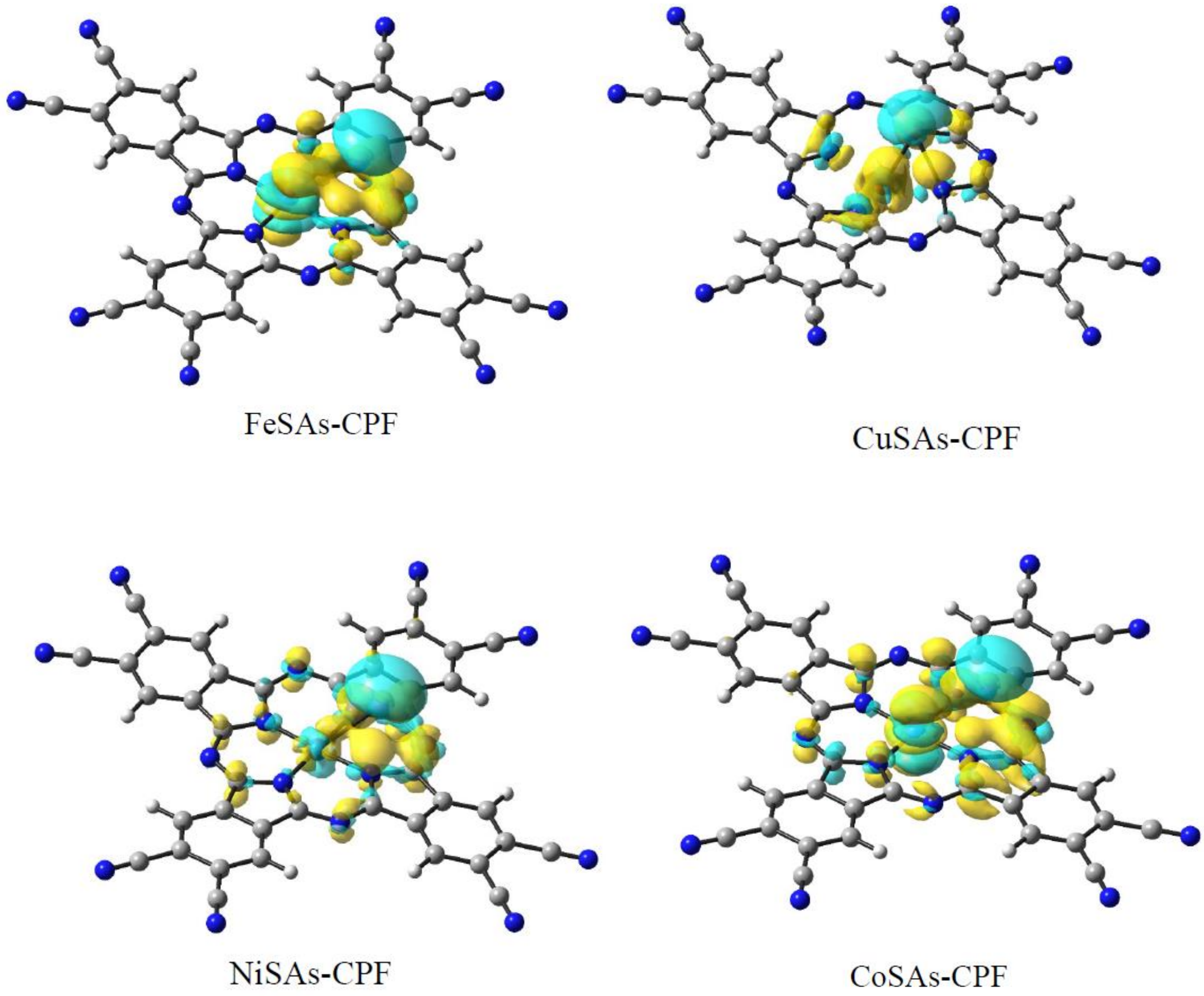

Figure S1. Electron density differences of a Li atom and the different MSAs-CPF surfaces, in which cyan and yellow regions indicate electron depletion and accumulation, respectively.

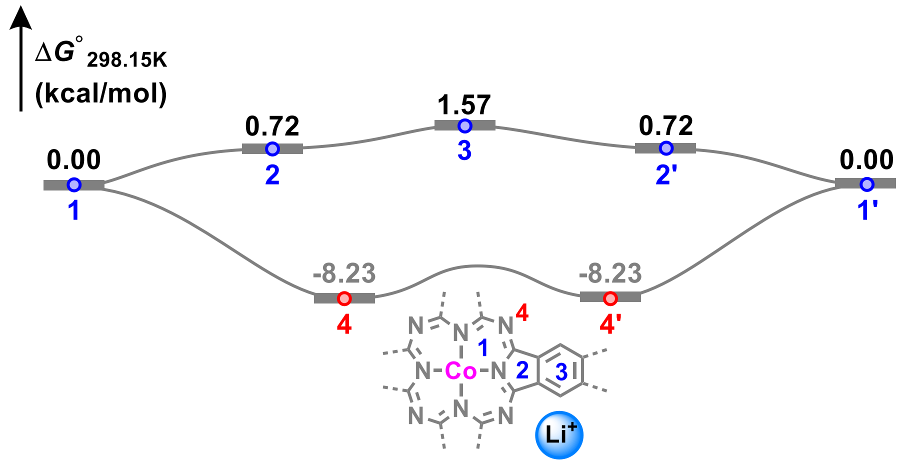

Figure S2. Gibbs energy profiles of the intermediate structures of Li^+ migration process at CoSAs-CPF surfaces.

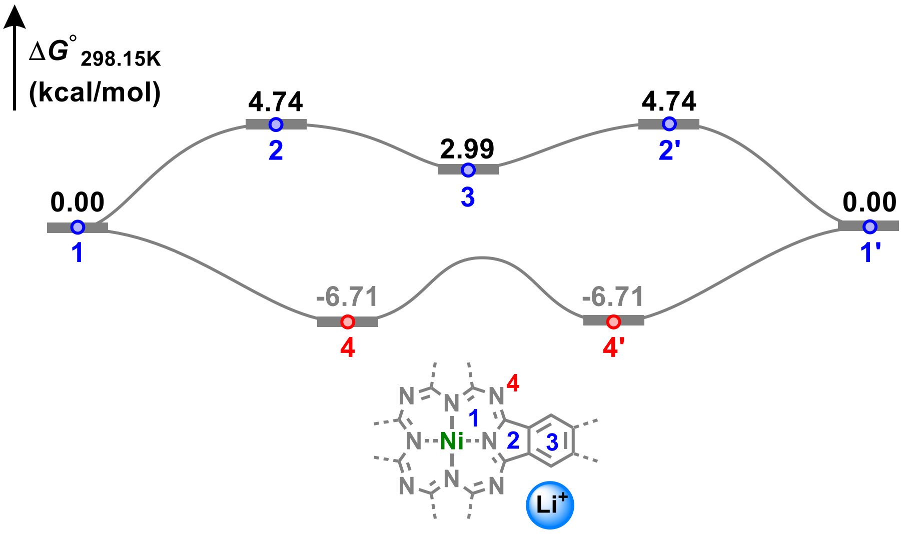

Figure S3. Gibbs energy profiles of the intermediate structures of Li^+ migration process at NiSAs-CPF surfaces.

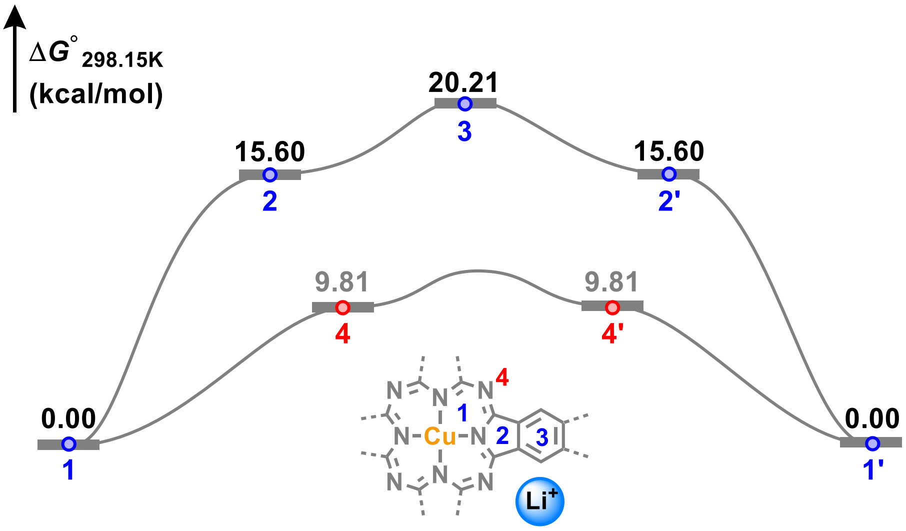

Figure S4. Gibbs energy profiles of the intermediate structures of Li^{+} migration process at CuSAs-CPF surfaces.

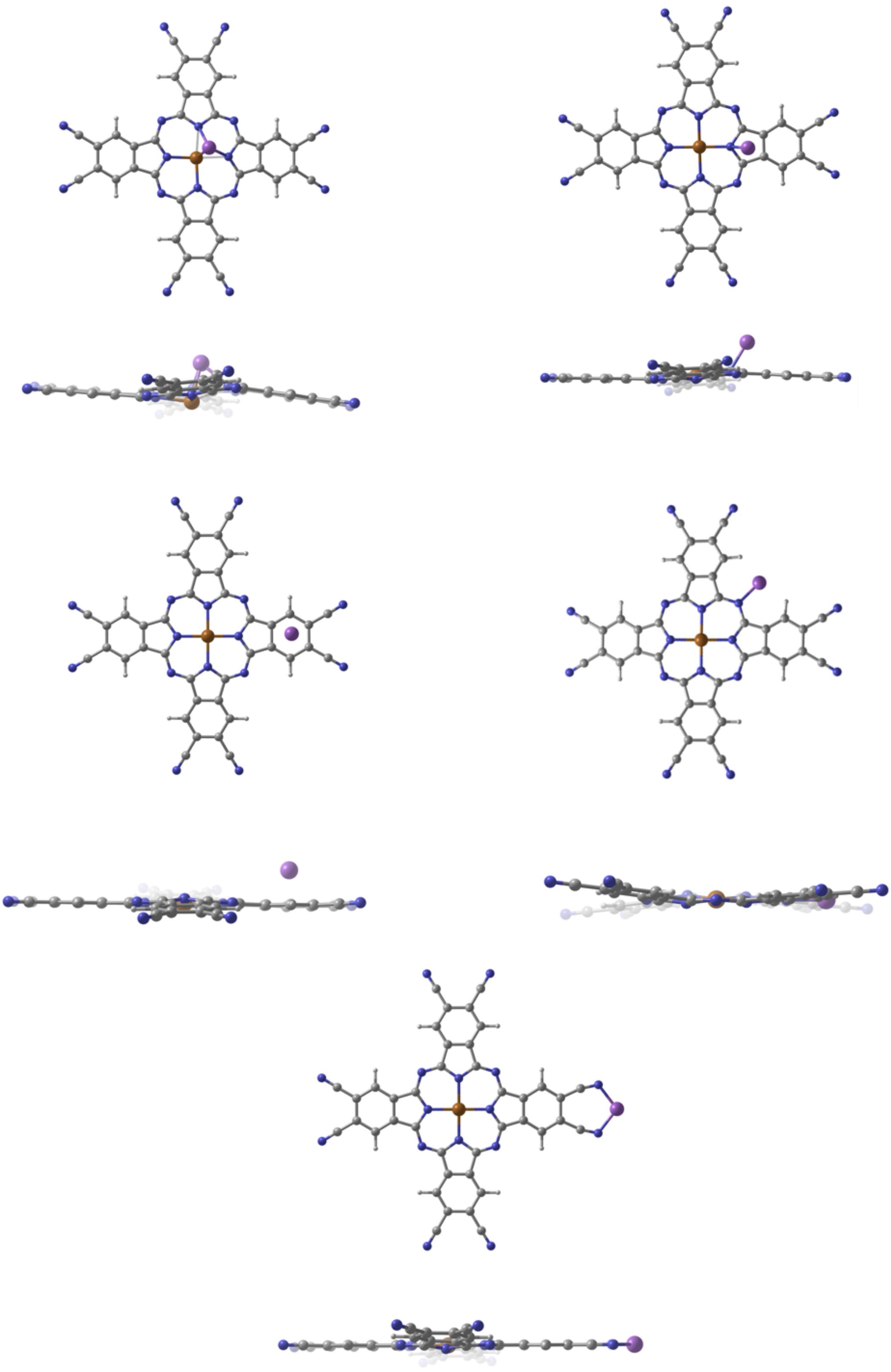

Figure S5. The top view and side view of the intermediate structures of Li^+ migration process at the CuSAs-CPF surface.

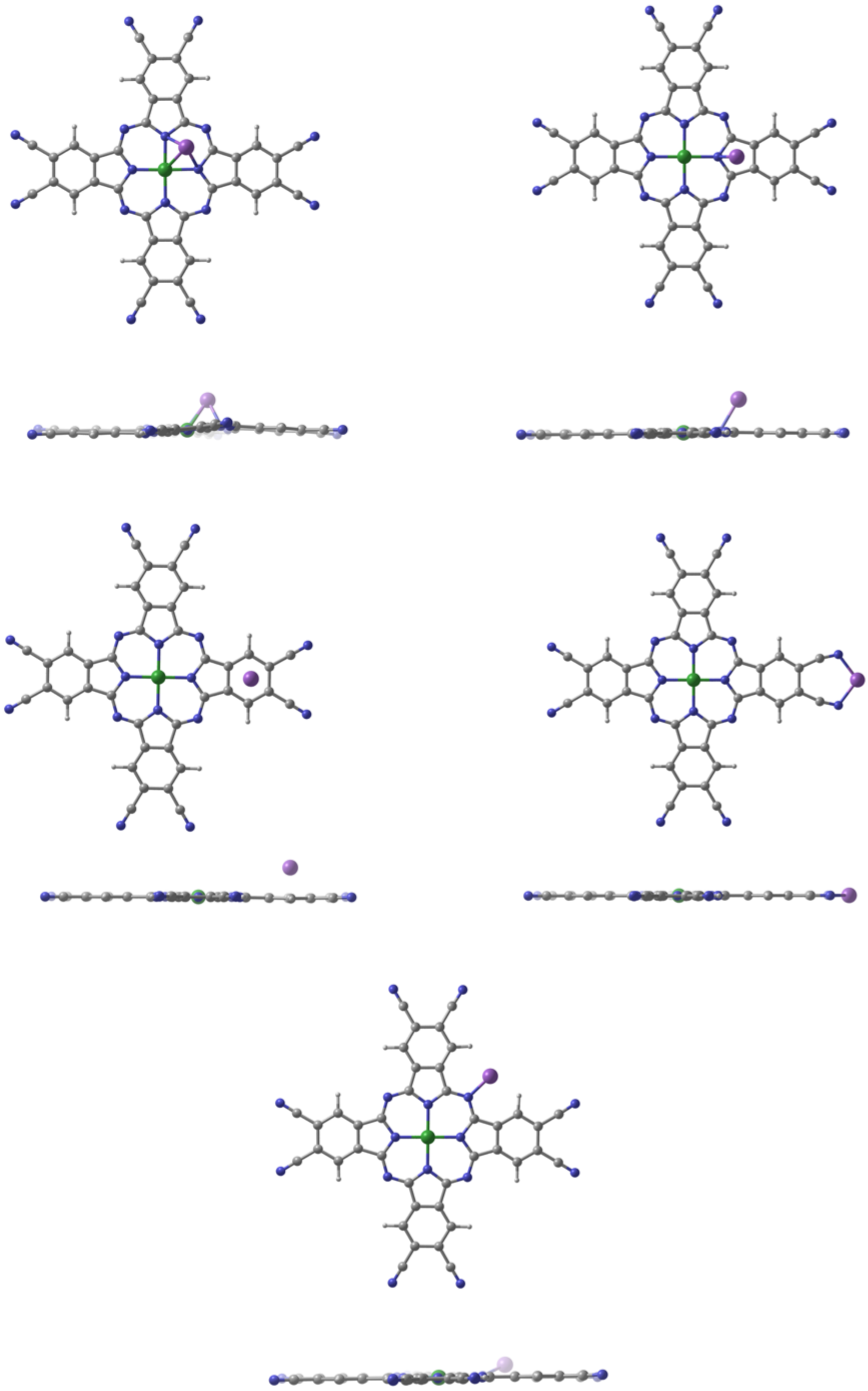

Figure S6. The top view and side view of the intermediate structures of Li^+ migration process at the NiSAs-CPF surface.

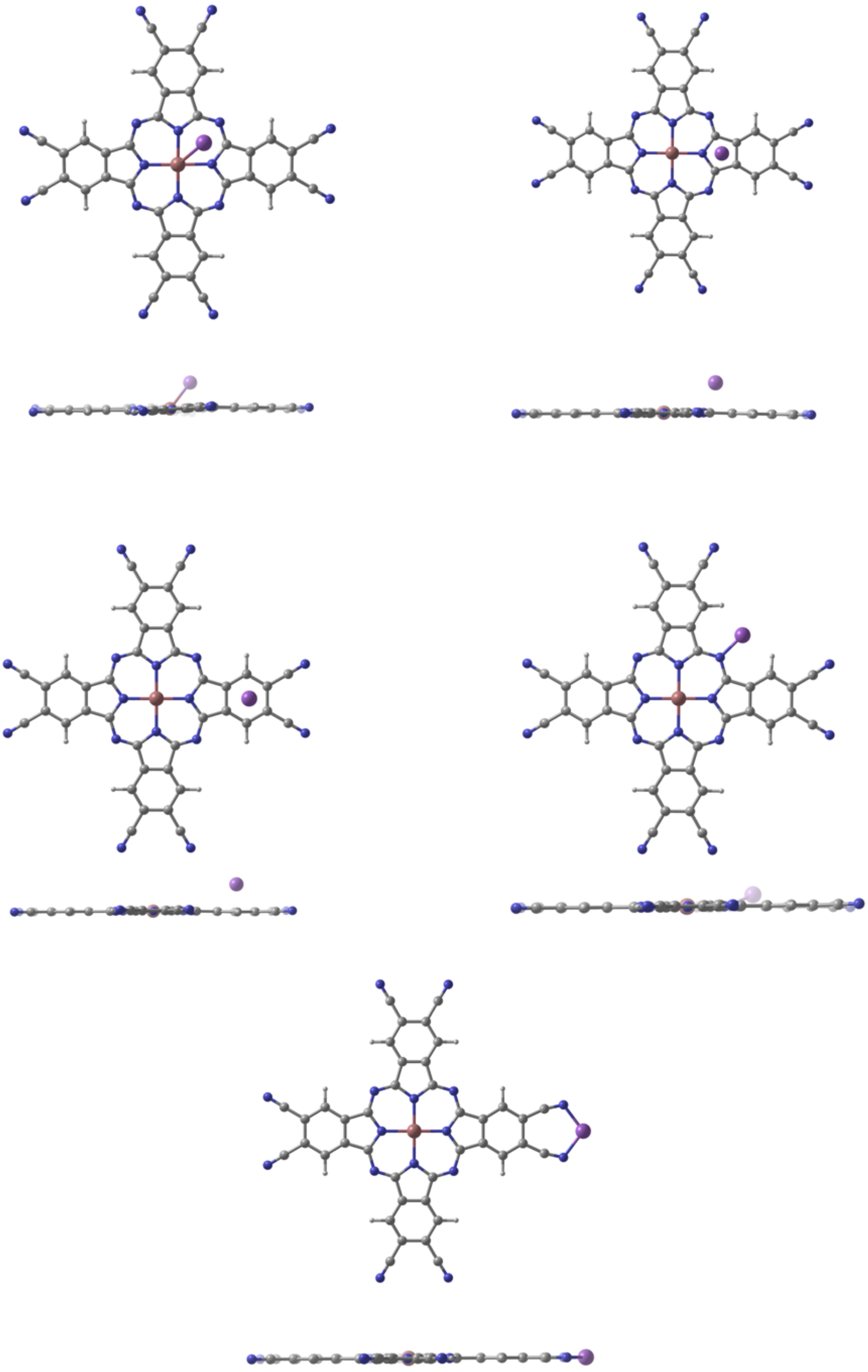

Figure S7. The top view and side view of the intermediate structures of Li^+ migration process at the CoSAs-CPF surface.

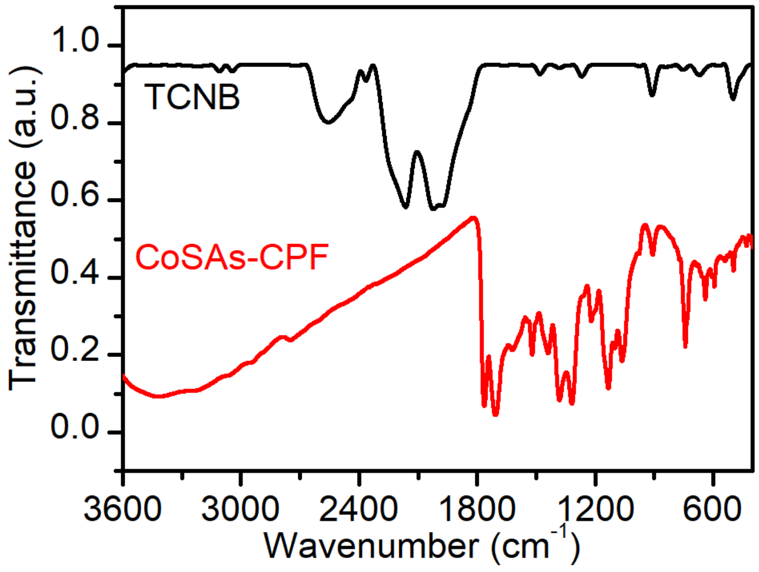

Figure S8. FT-IR of TCNB, CoSAs-CPF.

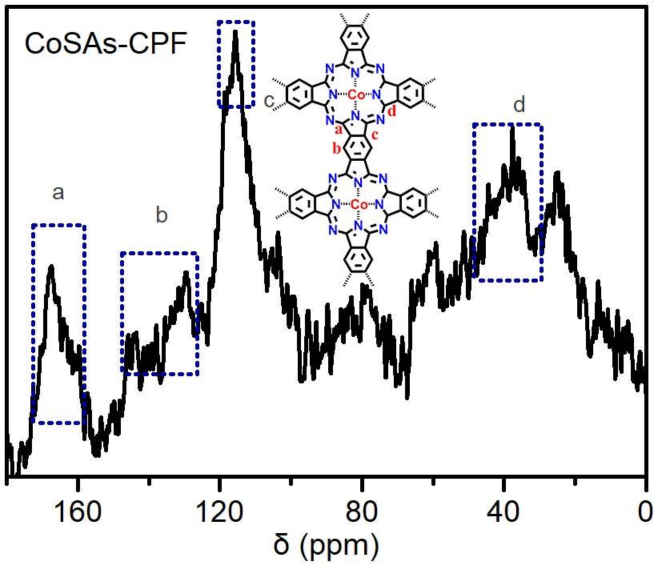

Figure S9. ^{13}C NMR of CoSAs-CPF.

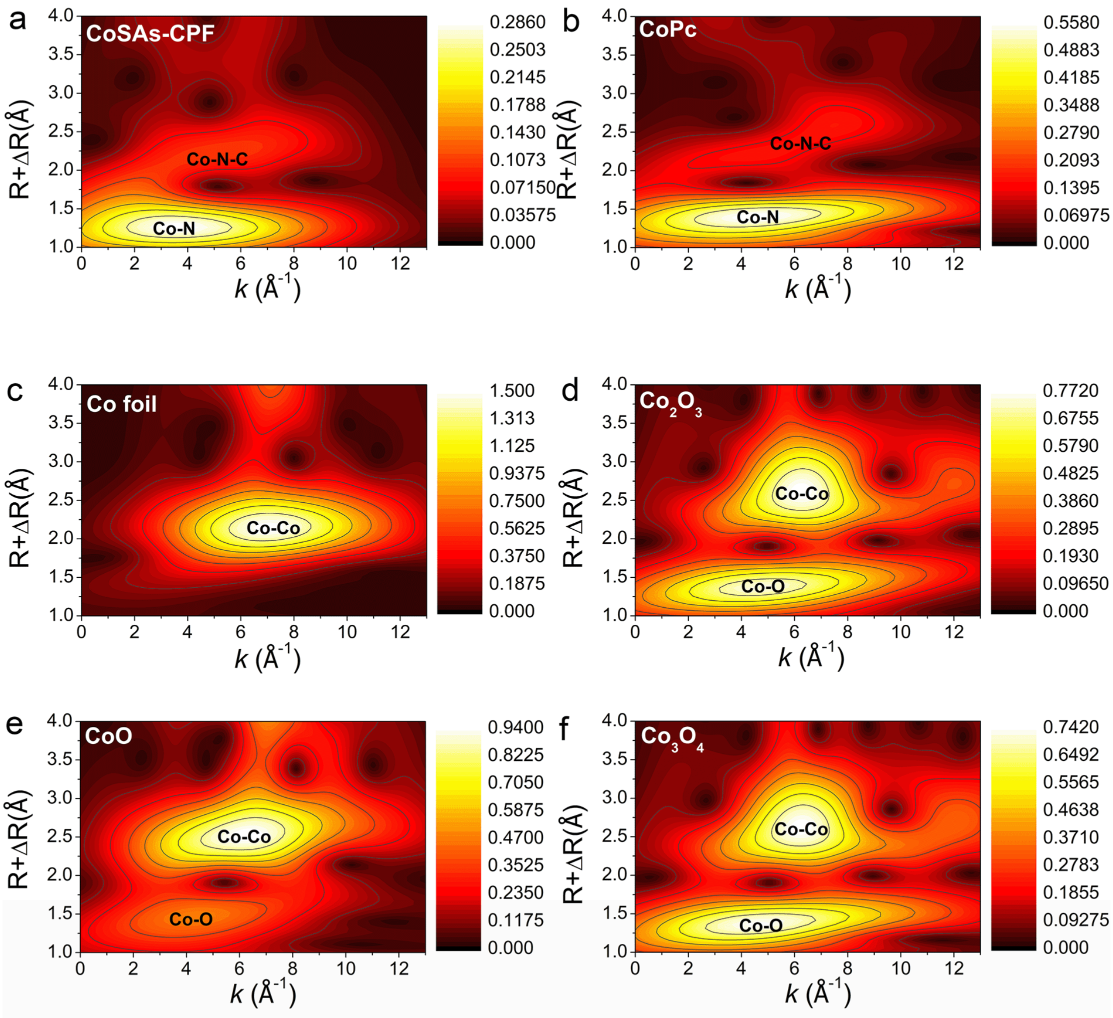

Figure S10. The wavelet transforms of χ(k) spectra of CoSAs-CPF, CoPc, Co foil, Co_2O_3, Co_3O_4.

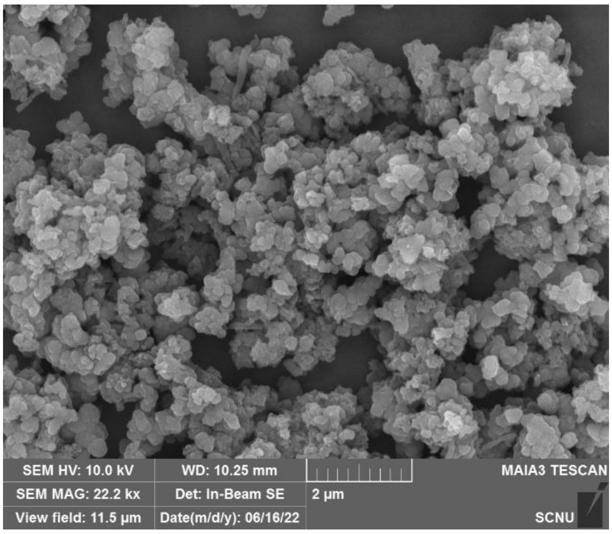

Figure S11. Scanning electron microscope (SEM) of CoSAs-CPF.

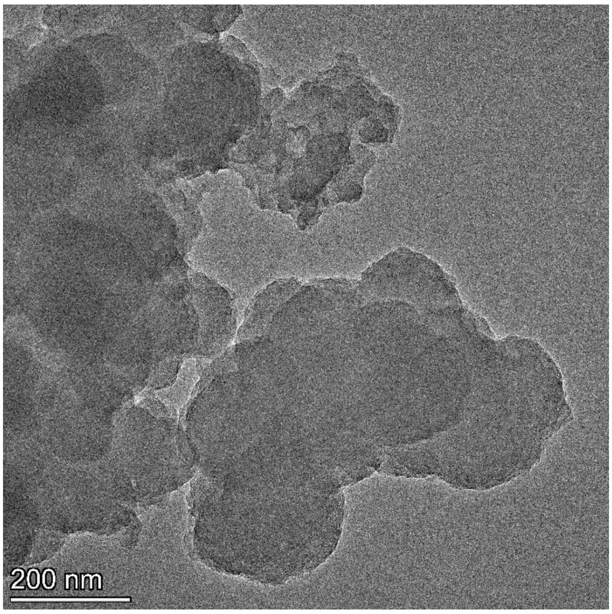

Figure S12. Transmission electron microscope (TEM) of CoSAs-CPF.

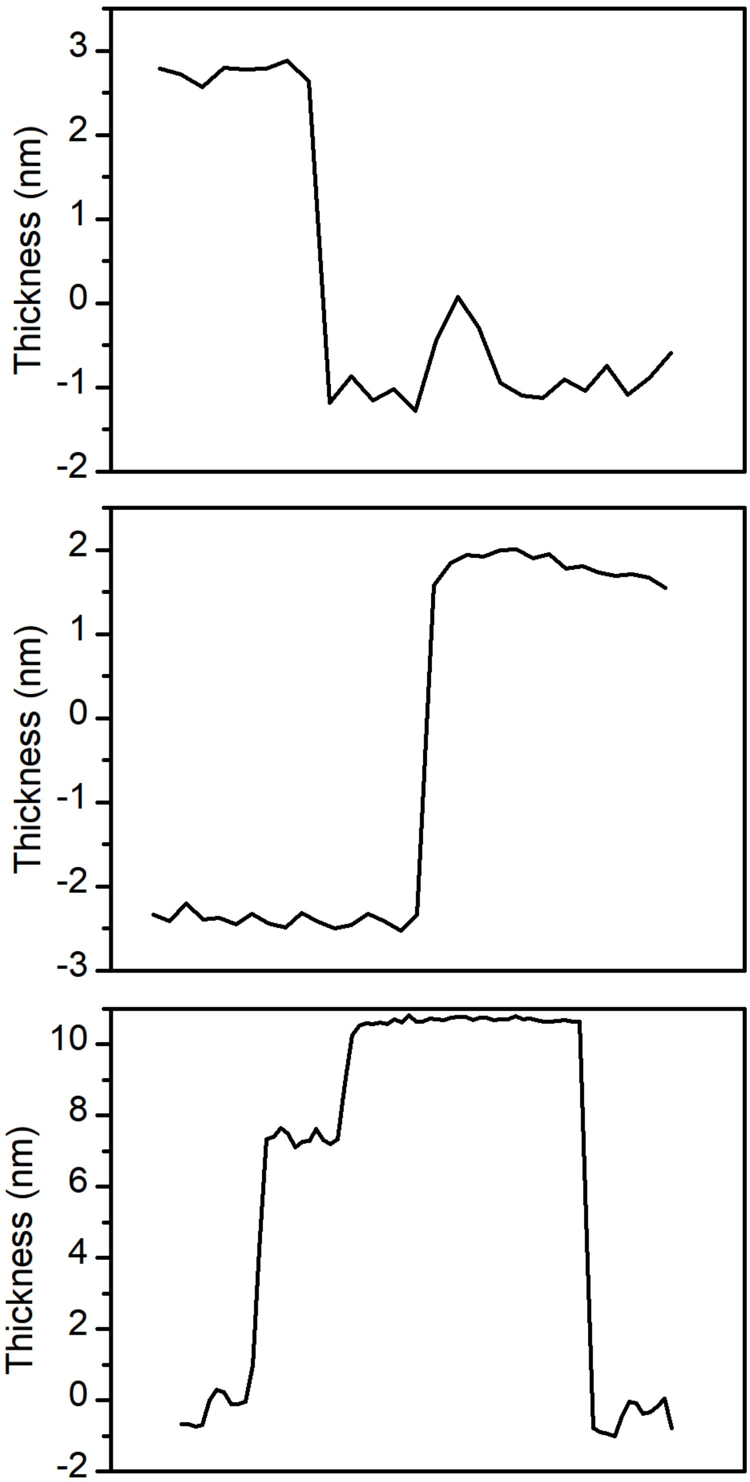

Figure S13. The thickness of CoSAs-CPF.

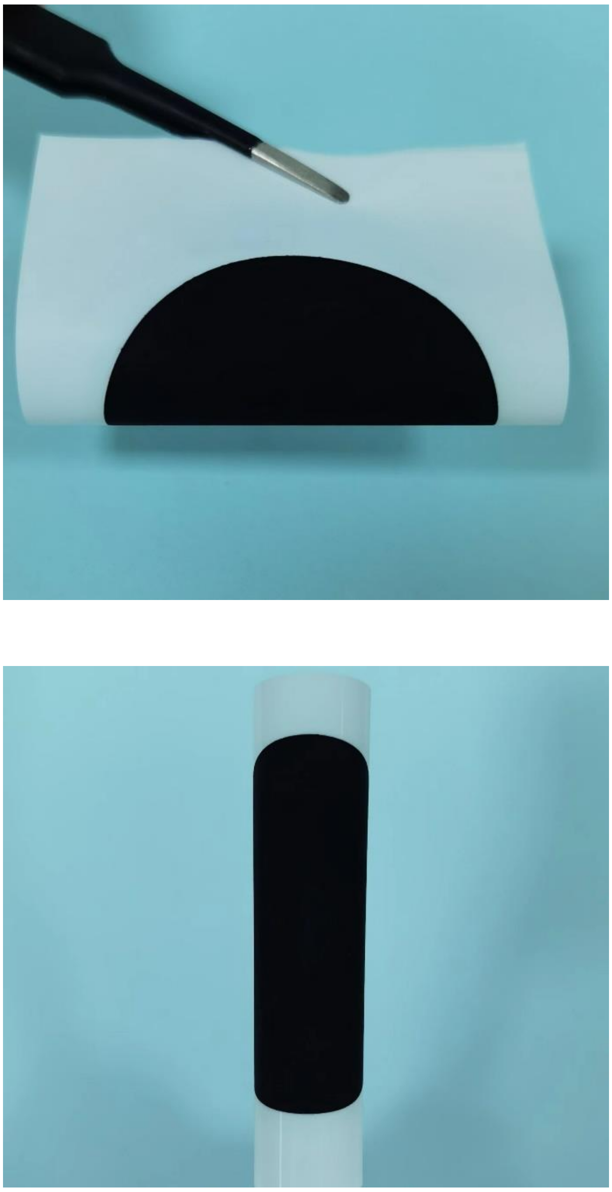

Figure S14. The optical photographs of CoSAs-CPF@PP.

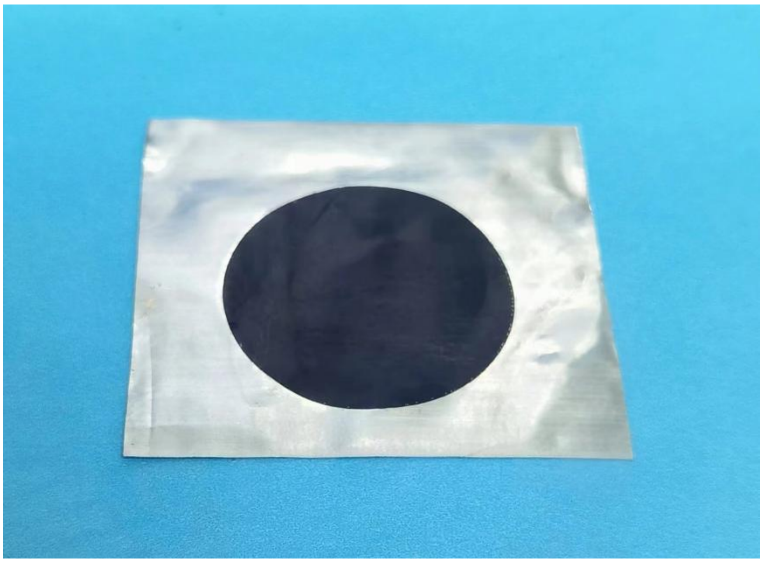

Figure S15. The optical photograph of artificial CoSAs-CPF SEI.

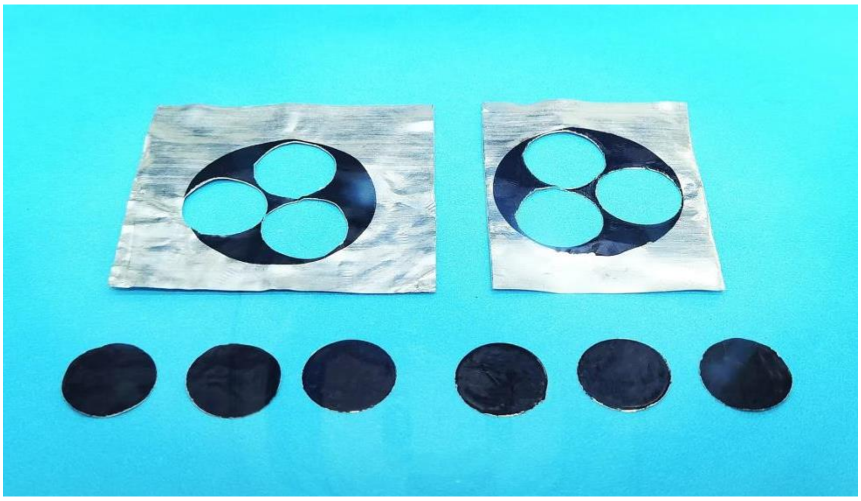

Figure S16. The optical photograph of CoSAs-CPF@Li which was cut into small discs with a diameter of 16 mm.

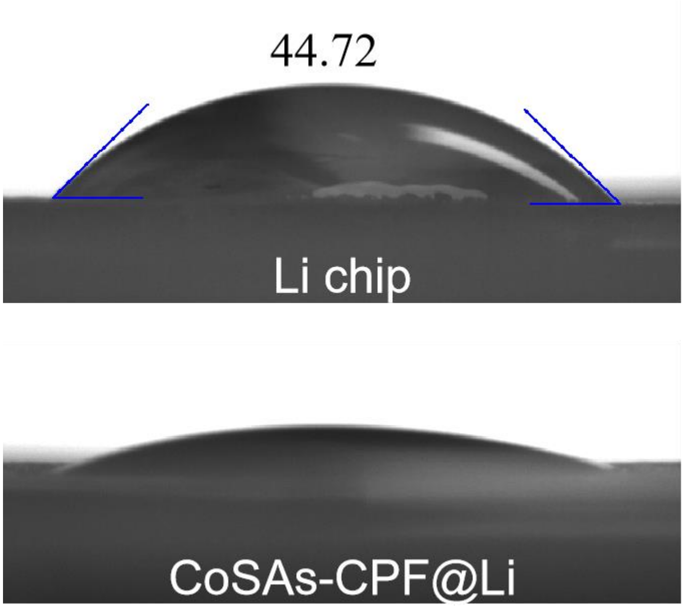

Figure S17. The contact angle of Li chip and CoSAs-CPF@PP.

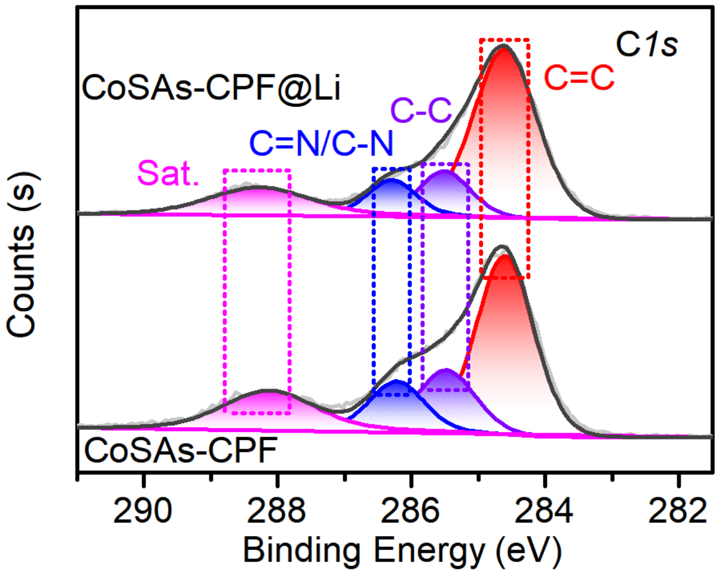

Figure S18. High-resolution C1s spectra of CoSAs-CPF and CoSAs-CPF@Li.

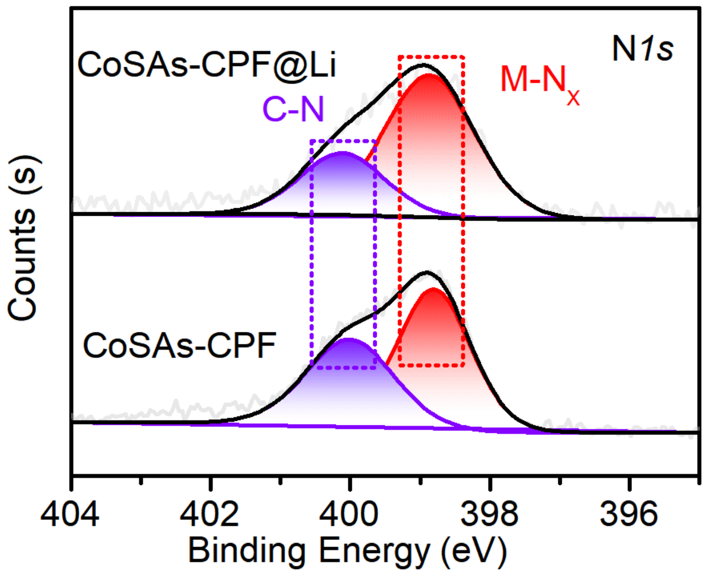

Figure S19. High-resolution N1s spectra of CoSAs-CPF and CoSAs-CPF@Li.

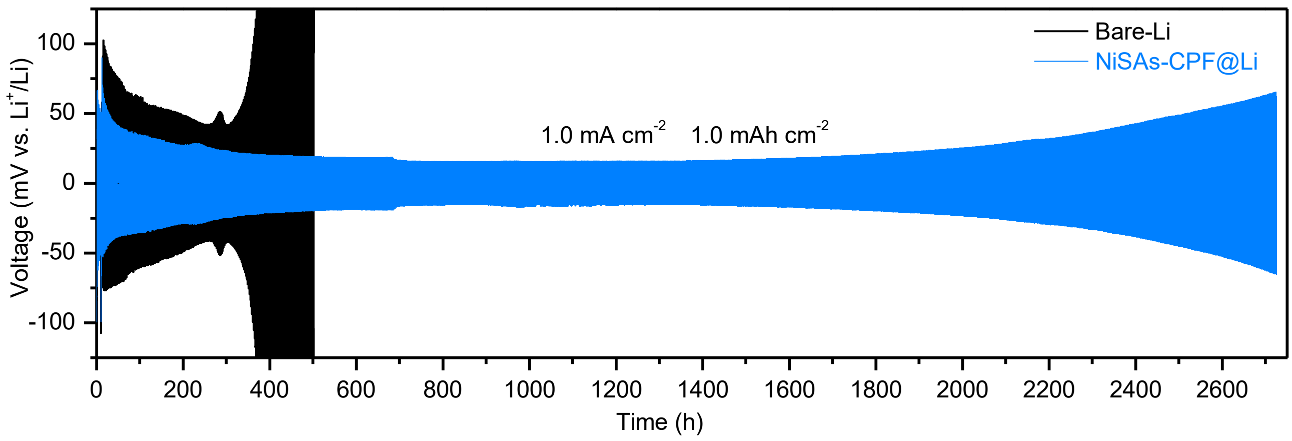

Figure S20. Cycling stability of bare-Li foil and NiSAs-CPF@Li symmetric cells.

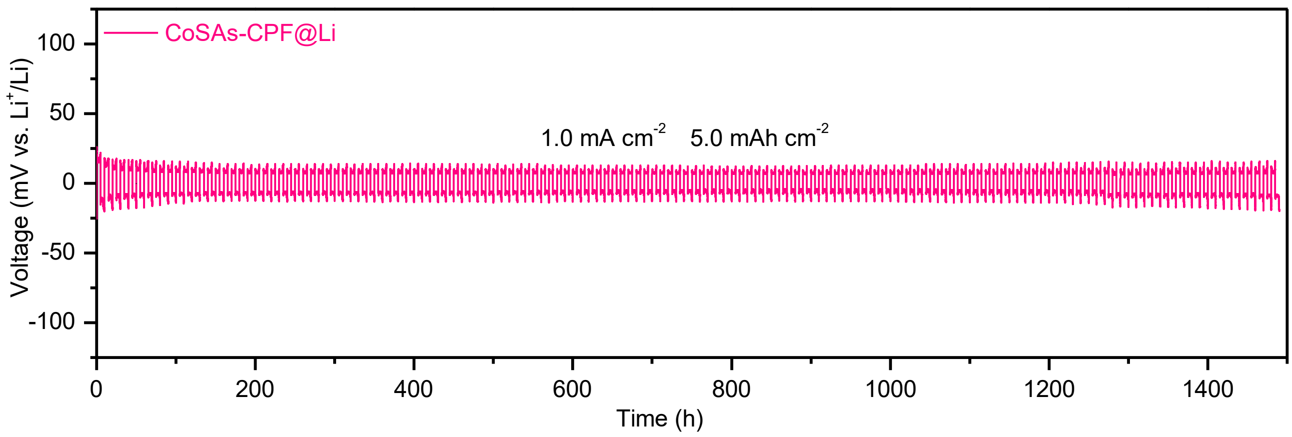

Figure S21. Galvanostatic cycling profiles of CoSAs-CPF modified Li||Li symmetric batteries with a current density of 1.0 mA cm^{-2} and areal capacity of 5.0 mAh cm^{-2}.

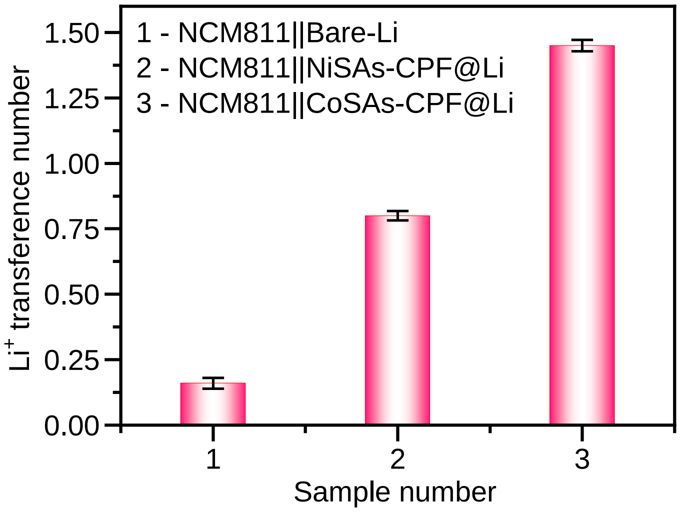

Figure S22. The average PPVs of NCM811||Bare-Li, NCM811||NiSAs-CPF@Li and NCM811||CoSAs-CPF@Li.

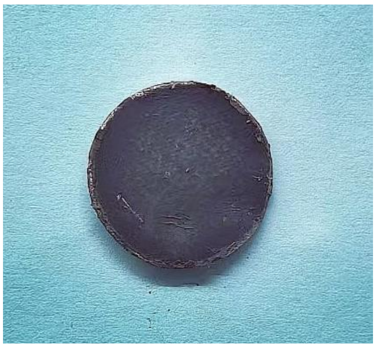

Figure S23. Li anode of disassembled NCM811||Bare-Li cell after cycling.

Table S1. Performance comparison of cells with LFP cathodes.

Anodes	Cathode Loading (mg cm^{-2})	Intial capacity (mAh g^{-1})	Cycling Rate	Cycle number	References
**CoSAs-CPF**	**30**	**133**	**1.0**	**850**	**Our work**
**CoSAs-CPF**	**20**	**144**	**1.0**	**1000**	**Our work**
sp^2c-COF	4	149.5	1	500	*7*
COF-LZU1	3.8	132	0.5	200	*8*
Tp-PaSO_3Li-COF	1.25	152	1.0	20	*9*
COF based QSSE	1.6	119.7	0.5	110	*10*
G@COF-1	8.8	~135	1.0	60	*11*
Fib-COF	2.2	~135	0.5	100	*12*
TpTt	3	150	0.5	450	*13*
LPC-2	1.25	144.6	0.2	35	*14*
TpPa-2SO_3H	2.8	128.8	1.0	200	*15*
g-C_3N_4/graphene/g-C_3N_4	20	175	0.3	180	*16*
Dynamic gel as artificial SEI	20	138	0.5	100	*17*
Diamond-like carbon coated PP	1.5	90	5.0	1000	*18*
$EMoS_2$	20	160	1.0	1000	*19*

Table S2. Performance comparison of cells with NCM811 cathodes.

**Anodes**	**Cathode Loading (mg cm^{-2})**	**Intial capacity (mAh g-1)**	**Cycling Rate**	**Cycle number**	**References**
**CoSAs-CPF**	**10**	**185**	**1**	**800**	**Our work**
**CoSAs-CPF**	**20**	**170**	**1**	**450**	**Our work**
COF SSE	2	~160	0.1	400	*20*
3D Lithiophilic Mo_2N-Modified Carbon Nanofiber	10	181	1/3	150	*21*
Nanodiamond particle (ND)-embedded SEI	21.5	186	0.5	112	*22*
Conjugated microporous thermosetting polymer@Li	~20	~200	/	150	*23*
LiF-rich SEI	25	~200	/	50	*24*
Hard carbon Li host	~10	~210	10 (mA cm^{-2})	200	*25*
Ethoxy(pentafluoro) cyclotriphosphazene as additive	30	200	0.5	50	*26*
Upgrading carbonate electrolytes with $LiNO_3$	20	200	0.5	150	*27*
$EMoS_2$	20	203	0.5	305	*19*

Table S3. Specifications of NCM811||Li pouch cell.

Cell component	Specification	Areal parameters	Total parameters
NCM811 cathode	Active material mass loading (95 wt%, 20 mg cm^{-2} each side)	21.05 mg cm^{-2}	7.90 g
Al current	Each side (mg cm^{-2}) (59 mm × 79 mm)	4 mg cm^{-2}	0.75 g
Electrolyte	Weight (mg cm^{-2}) Electrolyte/Capacity = (2.5 g Ah^{-1})	/	4.18 g
Anode (Li)	Thickness (100 μm) (62 mm × 82 mm)	5.34 mg cm^{-2}	2.17 g
Artificial SEI	CoSAs-CPF (62 mm × 82 mm)	0.5 mg cm^{-2}	0.20 g
Total	Weight (g)	/	15.20 g
Capacity	Capacity (Ah)	1.67 Ah	
Mid-value voltage	Voltage (V)	3.83 V	
**Energy density**	**(Wh kg^{-1})**	**421 Wh kg^{-1} Core of pouch cell**	

Energy density = Discharge capacity (Ah) × Mid-value voltage (V) / Total weight (kg).

Reference

1. G. W. T. H. B. S. G. E. S. M. J. Frisch, J. R. C. G. S. V. B. M. A. Robb, H. N. X. L. M. C. A. V. M. G. A. Petersson, B. G. J. R. G. B. M. H. P. H. J. Bloino, A. F. I. J. L. S. D. W.-Y. J. V. Ortiz, F. L. F. E. J. G. B. P. A. P. F. Ding, D. R. V. G. Z. J. G. N. R. T. Henderson, W. L. M. H. M. E. K. T. R. F. G. Zheng, M. I. T. N. Y. H. O. K. H. N. J. Hasegawa, K. T. J. A. M. J. J. E. P. T. Vreven, M. J. B. J. J. H. E. N. B. K. N. K. F. Ogliaro, V. N. T. A. K. R. K. J. N. Staroverov, A. P. R. J. C. B. S. S. I. K. Raghavachari, M. C. J. M. M. M. K. C. A. R. C. J. Tomasi, R. L. M. K. M. O. F. J. W. Ochterski, J. B. Foresman, Fox, D. J. *Gaussian 16, Revision C.01, Gaussian, Inc., Wallingford CT,* **2019**.
2. F. Weigend, R. Ahlrichs, Balanced basis sets of split valences, triple zeta valence and quadruple zeta valence quality for H to Rn: Design and assessment of accuracy. *Phys Chem Chem Phys* **2005**, *7*, 3297-305.
3. P. J. Stephens, F. J. Devlin, C. F. Chabalowski, M. J. Frisch, Ab Initio Calculation of Vibrational Absorption and Circular Dichroism Spectra Using Density Functional Force Fields. *J. Phys. Chem.* **1994**, *98*, 11623-11627.
4. S. Grimme, S. Ehrlich, L. Goerigk, Effect of the Damping Function in Dispersion Corrected Density Functional Theory. *J. Comput. Chem.* **2011**, 32, 1456-1465.
5. M. Xiao, T. Lu, Generalized Charge Decomposition Analysis (GCDA) Method. *Journal of Advances in Physical Chemistry* **2015**, *4*, 111-124.
6. T. Lu, F. W. Chen, Multiwfn: A multifunctional wavefunction analyzer. *J. Comput. Chem*. **2012**, *33,* 580-592.
7. C. H. Zhang, Y. X. Yang, Y. J. Sun, L. Y. Duan, Z. Y. Mei, Q. An, Q. Jing, G. F. Zhao, H. Guo, *Sci. China Mater.* **2023**, *66*, 2591-2600.
8. H. Y. Xie, Q. Hao, H. C. Jin, S. Xie, Z. W. Sun, Y. D. Ye, C. H. Zhang, D. Wang, H. X. Ji, L. J. Wan, *Sci. China Chem.* **2020**, *63*, 1306-1314.
9. J. Li, F. Q. Zhang, F. L. Li, Z. Z. Wu, C. L. Ma, Q. C. Xu, P. F. Wang, X. M. Zhang, *Chem. Commun.* **2020**, *56*, 2747-2750.
10. Z. N. Wang, W. Z. Zheng, B. H. Li, W. Z. Sun, L. Zhao, W. K. Yuan, *Chem. Eng. J.* **2022**, *433*, 133749.
11. Y. W. Song, P. Shi, B. Q. Li, X. Chen, C. X. Zhao, W. J. Chen, X. Q. Zhang, X. Chen, Q. Zhang, *Matter* **2021**, *4,* 253-264.
12. X. Y. Fan, Y. T. Zhang, Y. Y. Dou, X. D. Li, Z. Y. Zhao, X. J. Zhang, H. X. Wu, S. L. Qiao, *ACS Appl. Mater. Interfaces* **2023**, 1*5*, 51694-51703.
13. Z. H. Li, W. Y. Ji, T. X. Wang, Y. R. Zhang, Z. Li, X. S. Ding, B. H. Han, W. Feng, *ACS Appl. Mater.*

Interfaces **2021**, *13,* 22586-22596.

14. Z. Z. Wu, Q. C. Xu, J. Li, X. M. Zhang, *Chem. Eur. J.* **2021**, *27*, 4583-4587.
15. C. Wang, W. Z. Li, Y. H. Jin, J. B. Liu, H. Wang, Q. Q. Zhang, *Small* **2023**, *19,* 2300023.
16. P. Zhai, T. Wang, H. Jiang, J. Wan, Y. Wei, L. Wang, W. Liu, Q. Chen, W. Yang, Y. Cui, Y. Gong, *Adv. Mater*. **2021**, 33, 2006247.
17. C. Chen, J. Zhang, B. Hu, Q. Liang, X. Xiong, *Nat. Commun*. **2023**, 14, 4018.
18. Z. Li, M. Peng, X. Zhou, K. Shin, S. Tunmee, X. Zhang, C. Xie, H. Saitoh, Y. Zheng, Z. Zhou, Y. Tang, *Adv. Mater*. **2021**, 33, 2100793.
19. J. Qin, F. Pei, R. Wang, L, Wu, Y. Han, P. Xiao, Y. Shen, L. Yuan, Y. Huang, D. Wang, *Adv. Mater*. **2024**, 10.1002/adma.202312773.
20. C. Q. Niu, W. J. Luo, C. M. Dai, C. B. Yu, Y. X. Xu, *Angew. Chem. Int. Ed*. **2021**, *60*, 24915-24923.
21. L. Luo, J. Li, H. Yaghoobnejad Asl, A. Manthiram, *Adv. Mater.* **2019**, 31, 1904537.
22. Z. Shen, W. Zhang, S. Li, S. Mao, X. Wang, F. Chen, Y. Lu, *Nano Lett.* **2020**, 20, 6606.
23. K. Zhang, W. Liu, Y. Gao, X. Wang, Z. Chen, R. Ning, W. Yu, R. Li, L. Li, X. Li, K. Yuan, L. Ma, N. Li, C. Shen, W. Huang, K. Xie, K. P. Loh, *Adv. Mater.* **2021**, 33, 2006323.
24. L. Lin, L. Suo, Y. s. Hu, H. Li, X. Huang, L. Chen, *Adv. Energy Mater.* **2021**, 11, 2003709.
25. H. Gong, Y. Chen, S. Chen, C. Xu, Y. Yang, Y. Ye, Z. Huang, R. Ning, Y. Cui, Z. Bao, *ACS Energy Lett*. **2022**, 7, 4417.
26. L. Zhang, F. Min, Y. Luo, G. Dang, H. Gu, Q. Dong, M. Zhang, L. Sheng, Y. Shen, L. Chen, J. Xie, *Nano Energy* **2022**, 96, 107122.
27. Q. Zhao, N. W. Utomo, A. L. Kocen, S. Jin, Y. Deng, V. X. Zhu, S. Moganty, G. W. Coates, L. A. Archer, *Angew. Chem., Int. Ed*. **2022**, 61, 202116214.
